# Medical College Note

**Published:** 1895-07

**Authors:** 


### Medical College Note.

JEFFERSON MEDICAL COLLEGE held its sixty-ninth annual commencement in Philadelphia, May 15, 1895. The class numbered 148, and it is reported that nearly one-fourth of the members were plucked at the final examination. An explanation is offered that this may be taken as a compliment to the state medical examining and licensing board.

